# IMPACT OF SEX AND ADRD ON DAYS SPENT AT HOME AFTER HIP FRACTURE AMONG OLDER MEDICARE BENEFICIARIES

**DOI:** 10.1093/geroni/igad104.1332

**Published:** 2023-12-21

**Authors:** Denise Orwig, Heather Mutchie, Ann Gruber-Baldini, Abree Johnson, Jay Magaziner, Jason Falvey

**Affiliations:** University of Maryland School of Medicine, Baltimore, Maryland, United States; University of Maryland, Baltimore, Maryland, United States; University of Maryland School of Medicine, Baltimore, Maryland, United States; University of Maryland, Baltimore, Maryland, United States; University of Maryland Baltimore, Baltimore, Maryland, United States; University of Maryland School of Medicine, Baltimore, Maryland, United States

## Abstract

Men recovering from hip fracture tend to have poorer health and outcomes compared to women. Alzheimer’s disease or related dementias (ADRD), which is differentially recognized between the sexes, is one potential factor contributing to these differences. Data were drawn from 2010-2017 Medicare fee-for-service beneficiaries aged 65 years and older who survived hospitalization for hip fracture. Our primary outcome was days alive and at home (DAAH) which subtracts from 365 the number of days in a hospital and/or rehabilitation facility, nursing home, emergency department or hospital observation, and days not alive (index fracture admission day to date of death, if applicable). Multivariable Poisson regressions were adjusted for demographics, injury severity, chronic disease burden, and hospital-level fixed effects, to model the association between DAAH and ADRD in the 12 months post hip fracture, with an interaction term for sex and ADRD status. Among survivors, males with ADRD spent a mean of 160.7 DAAH compared to 228.4 for males without ADRD, 177.8 for females with ADRD and 248.0 for females without ADRD. In adjusted analyses, males without ADRD spent 8.2% fewer DAAH compared to females (rate ratio (RR)=0.92, 95% CI 0.92-0.92). This sex difference increased significantly when comparing those living with ADRD, with males spending an additional 3.3% fewer DAAH (interaction RR=0.96, 95% CI 0.96-0.97). Males spend fewer DAAH after hip fracture than females and males with ADRD have the fewest DAAH. This suggests that cognitive impairment may be a small but significant contributor to sex-based differences observed during hip fracture recovery.

